# Mediator Role of Dissociative Experiences in the Effect of Childhood Traumas on Emotion Regulation Difficulty and Parental Child-Containing Function

**DOI:** 10.3390/children11060618

**Published:** 2024-05-22

**Authors:** Elif Yöyen, Fatih Bal, Tülay Güneri Barış, Meryem Selva Arslan, Gülşen Filazoğlu Çokluk

**Affiliations:** 1 Faculty of Humanities and Social Sciences, Department of Psychology, Sakarya University, 54050 Sakarya, Turkey; fatihbal@sakarya.edu.tr; 2 Institute of Business Administration, Department of Health Sciences, Sakarya University, 54050 Sakarya, Turkey; 3 Institute of Social Sciences, Department of Clinical Psychology, Marmara University, 34722 İstanbul, Turkey; selvarslan@gmail.com; 4 Faculty of Economics, Administrative and Social Sciences, Istanbul Gelişim University, 34310 İstanbul, Turkey

**Keywords:** childhood traumas, dissociation, difficulty in emotion regulation, parental child-containing function

## Abstract

The aim of this study is to examine the mediating role of dissociation in emotional regulation and parental child-containing function skills of mothers exposed to childhood trauma. The research was conducted with 400 mothers between the ages of 20–60 who had childhood trauma and currently have children between the ages of 0–18. The relational screening model, one of the general screening models, was used in the research. The sample of the research was selected using the convenient sampling method and the simple random method. Research data were collected with the Sociodemographic Information Form, Childhood Trauma Scale (CTS), Parental Child-Containing Function Scale (PCCFS), Emotion Regulation Difficulty Scale (ERDS), and Dissociative Experiences Scale (DES). According to the results obtained in the study, physical abuse (β = 0.197; 95% CI [0.124; 0.268]), physical neglect (β = 0.232; 95% CI [0.161; 0.306]), emotional abuse (β = 0.238; 95% CI [0.169; 0.309]), emotional neglect (β = 0.210; 95% CI [0.150; 0.275]), and sexual abuse (β = 0.139; 95% CI [0.058; 0.220]) were found to have a significant indirect effect on emotion regulation difficulties through dissociative experiences. In addition, physical abuse (β = 0.122; 95% CI [0.071; 0.181]), physical neglect (β = 0.151; 95% CI [0.084; 0.228]), emotional abuse (β = 0.158; 95% CI [0.086; 0.238]), emotional neglect (β = 0.159; 95% CI [0.093; 0.235]), and sexual abuse (β = 0.086; 95% CI [0.039; 0.150] was found to have a significant indirect effect on parental child-containing function skills through dissociative experiences.

## 1. Introduction

There are different definitions of childhood trauma in the literature. According to the definition of the World Health Organization (WHO) in 2016, childhood trauma is behavior that constitutes actual or potential harm to the child’s health, survival, development, or dignity, existing in the context of a responsible, trustworthy, or power-based relationship. These behaviors include any form of physical and/or emotional mistreatment, sexual abuse, neglect or indifference, or any form of commercial or other exploitation [1]. Childhood traumas include two situations: abuse and neglect. While abuse is behavior that will disrupt children’s emotional, mental, physical, and social development, neglect can be defined as depriving children of their needs for care, love, nutrition, and education. Scientific studies emphasize that child abuse and neglect can have serious, harmful short- and long-term effects on children’s cognitive, socio-emotional, and behavioral development. It has been noted that abuse and neglect occurring early in life are associated with severe cognitive and academic deficits, social withdrawal and limited peer interactions, and internalizing (as opposed to externalizing) problems throughout later developmental periods, especially childhood and adolescence [2]. There is also evidence that childhood abuse and neglect are important in the development of psychopathology in adulthood. These pathologies include physical self-harm and suicidal behavior; antisocial personality disorder; other psychiatric diseases, especially mood and anxiety disorders; sleep disorders; nightmares; phobias; somatic complaints; fear response; attention deficit and hyperactivity disorder; secondary enuresis; amnesia in the early period of abuse; excessive fantasizing; trance-like states; sleepwalking; high rates of depression; post-traumatic stress disorder; somatization disorder; hypochondriasis; eating disorders; sexual dysfunction; borderline personality disorder; conversion disorder; developmental disorders; panic disorder; increased crime and violent behavior; and dissociative symptoms [3]

Dissociation, one of the consequences of childhood trauma, is the absence of normal integration of thoughts, feelings, and experiences into consciousness and memory. The emergence of dissociation occurs as a protective mechanism to protect the person from the intensity of the stressful event [4,5]. This protective mechanism functions as a necessity to adapt in order to cope with this process when the child is exposed to living in an abusive environment during the growth period [6]. Since the child loses his sense of trust in people, the child learns to live and develop by creating spaces where the child feels in control and strong in order to cope with the feeling of unpredictability the child feels in the face of abuse [7,8]. When the child who manages to survive in this way reaches adulthood, the child functions in a similar pattern in life and may use dissociative coping styles.

Traumas experienced in childhood cause not only dissociation but also the formation and distortion of personality, as it is a process in which development continues [9]. The effects of childhood trauma negatively impact the way peoples think, feel, and behave in adulthood. Among the most visible consequences of this are the attitudes a person exhibits when a person becomes a parent. It can be a challenging experience for an individual who bears the traces of abuse in childhood to demonstrate appropriate parental skills towards their own child in adulthood [10,11].

The parent’s past experiences, psychopathologies such as dissociation that develop in correlation with these experiences, and the parent’s personality, as well as the parent’s emotional regulation capacity, may be important factors in parenting skills. Emotion regulation skills significantly affect the way mothers care for their children and establish relationships [12]. By understanding the child’s needs correctly, a mother’s reactions when the child’s negative emotions are revealed are based on her own emotion regulation skills. Emotion regulation skills are the ability to recognize and understand emotions, accept emotions, control impulsive behaviors and behave in desired ways when experiencing negative emotions, and use appropriate emotion regulation strategies depending on the situation for situational and personal goals [13]. A mother who can recognize and understand her own emotions, accept her emotions, control her impulsive behaviors, and control her emotional reactions in order to achieve her personal goals can be defined as a mother who has appropriate emotion regulation skills. However, there are studies showing that people who were exposed to negative treatment in childhood have difficulties in regulating emotions, avoid emotional experiences, and have a low capacity to accept emotions [14,15].

Another factor that affects parenting skills is parental child-containing function skills. According to Bion, the baby, who records in his mind the nature of the mother–infant relationship that begins to form in infancy as an ‘internal design’, copes with the stimuli coming from the external and internal world while growing up, thanks to the inclusive function of the mother [16]. This function is realized thanks to the mother’s ability holding, as stated by Winnicott. When the mother feels, makes sense of and meets the baby’s needs, the child’s internal tension and needs are met and soothed [17]. In other words, in the relationship between mother and child, all of the functions that ensure that the mother’s general attitude and the quality of the relationship coincide with the child’s psychological needs are containing functions [18]. The containing function can be interpreted as the mother’s attitude towards her child in the attachment between mother and child. The mother’s attitude towards her child is important in the bonding process between mother and child. In children’s relationship with their parents, the parent’s inability to feel, understand, and transform the child’s needs and the inability to soothe the child’s emotions are considered psychopathology related to containing functionality [17]. According to research, it is suggested that the reason for the situation experienced by parents who feel inadequate in effective parenting skills is due to their own parents’ ineffective parenting and their emotional regulation capacity due to the neglect and abuse they experienced [19].

The aim of this study is to examine the mediating role of dissociation in the relationship between emotional regulation difficulties and parental child-containing functioning skills of mothers with childhood trauma experiences. The problem of the study is the following: Does dissociation play a mediating role between the emotional regulation skills and the parental child-containing function of mothers who were exposed to childhood trauma?

## 2. Materials and Methods

### 2.1. Procedure

The research began after obtaining approval from Marmara University Institute of Social Sciences Research Ethics Committee with the date 17 January 2022, number 2021-14 and protocol number 2021-3/26. The people who participated in the research took part on a voluntary basis. Research advertisements were randomly announced in online correspondence groups and participants were thus randomly selected. In order to increase the scientific value and generalizability of the research, sample selection was made with this method. However, the random selection of the sample remained limited to the social network accessible to the researchers. A consent form that did not contain private personal information from the participants, a short text about the content and purpose of the study, and the scales used in the research were presented. The administration of the scales took an average of 15 min.

### 2.2. Research Method

The PROCESS MACRO software (version 3) installed in the SPSS 25 Package program was used in the analyses. The “Bootstrap” method is used in the analysis of mediating variables performed with this method. In this method, sub-samples are randomly created from the research data and the analyzed mediation model is tested for these sub-samples, and the analysis results of the larger research sample and sub-samples are compared with each other [19]. In the current study, 5000 bootstrap samples were used during the mediator variable analysis, as suggested by Hayes [20]. In the steps suggested by Baron and Kenny, the fact that the lower and upper limit values of the confidence interval (LLCI; ULCI) do not contain the value of “0” and the results of the Sobel test are meaningful after the assumptions are met indicates that the effect between the variables is significant [20]. In cases where the mediating effect is significant, the mediating variable may explain some or all of the relationship between the independent and dependent variable. When the whole relationship is explained, it is defined as a full mediator, and when a part of it is explained, the variable is defined as a partial mediator. When full mediation occurs, the relationship between the dependent and independent variable is expected to become statistically insignificant when the mediating variable is added to the analysis. In partial mediation, the mediating variable explains a certain part of the relationship between the dependent and independent variable.

In this research, where the relational screening model was used, Ordinary Least Squares (OLS) was used for regression analyses. The purpose of using OLS analyses is to estimate regression coefficients. OLS finds the best coefficients by minimizing the sum of squares of errors. The aim of the research is to determine the intermediary role. In this context, regression models were used to determine the mediating role, and the necessary steps to test the mediating effect in the relationship between variables, as stated by Baron and Kenny, were implemented sequentially. After the regression models, the test developed by Sobel [21] was applied to determine the mediation effect, and the confidence intervals were examined. When looking at the final cut-off points to decide the presence of multicollinearity, the tolerance value of less than 0.10 and the Variance Inflation Factors (VIF) value of more than 0.10 show that the variables do not indicate multicollinearity. In the study, it is seen that the residual values for the analysis of extreme values are less than 16.27, which is the critical value for three variables. This finding shows that it does not exceed Mahalanobis distances and does not have extreme values. Tolerance and VIF values for the analyzes are presented in Table 1.

### 2.3. The Sample

The sample of the research consists of 400 mothers between the ages of 20–60 who have experienced childhood trauma and currently have children between the ages of 0–18. The demographic characteristics of the participants are presented in Table 1. When Table 1 is examined, there are 33 participants (8.3%) between the ages of 18–30, 200 participants (50%) between the ages of 31–40, 136 participants (34%) between the ages of 41–50, and 31 participants (7.8%) between the ages of 51–60. When the education status of the participants is examined, 21 participants (5.3%) were at primary school level, 16 participants (4%) were at secondary school level, 78 participants (19.5%) were at high school level, 207 participants (51.7%) were at university level, and 78 participants (19.5%) were at postgraduate/doctoral level. There are 4 participants (1%) whose marital status is single, 355 (88.8%) who are married, 36 (9%) who are divorced, 5 (1.3%) who have lost their spouse, and 4 (1%) who are cohabiting. In terms of employment status, 145 (36.3%) participants reported that they were housewives, 34 (8.5%) participants reported that they worked part-time, and 221 (55.3%) participants reported that they had a regular job. A total of 122 (30.5%) participants have one child, 193 (48.3%) participants have 2 children, 61 (15.3%) participants have 3 children, and 24 (5.9%) participants have 4 or more children. When describing their relationship with their child, 180 (45%) participants described it as very good, 188 (47%) participants described it as good, 30 (7.5%) participants described it as average, and 2 (0.5%) participants described it as bad. When the income level perceived by the participants was examined, 199 (49.7%) participants stated their income level as bad, 77 (19.3%) as medium, and 124 (31.0%) as good. A total of 172 (43%) participants had a history of receiving psychological support and 228 (57%) did not receive psychological support. Meanwhile, 213 (53.3%) participants had a migration history, and 187 (46.8%) participants did not have a migration history.

### 2.4. Data-Collection Tools

The scales used in the study are Sociodemographic Information Form, Childhood Trauma Scale (CTS), Dissociative Experiences Scale (DES), Emotion Regulation Difficulty Scale (ERDS), and Parental Child-Containing Function Scale (PCCFS)

#### 2.4.1. Sociodemographic Information Form

The Sociodemographic Information Form is a form prepared by the researchers, in which information such as age, education status, working status, marital status, and number of children are discussed with the participants.

#### 2.4.2. Childhood Trauma Scale (CTS)

The Childhood Trauma Scale is a five-point Likert-type self-report scale developed by Bernstein and his colleague in 1994 [22]. The scale items, which have 5 subscales as emotional abuse, physical abuse, sexual abuse, emotional neglect, and physical neglect, are scored between 1 and 5. The Turkish validity and reliability study of the scale was conducted by Şar and his colleague [23]. In this study, the Cronbach’s alpha value of the scale was calculated as 0.90.

#### 2.4.3. Emotion Regulation Difficulty Scale (ERDS)

The scale was developed by Bjureberg and et al. [24] from Gratz and Roemer’s [13] Emotion Regulation Difficulty scale. The aim of the scale is to comprehensively examine difficulties in emotion regulation, which have serious adverse effects on psychological health and functioning. The scale is a self-report list consisting of five subscales. The Turkish reliability and validity studies of the scale were carried out by Yiğit and Yiğit [25], and the Cronbach’s alpha value was found to be 0.92. In this study, the Cronbach’s alpha value of the scale was calculated as 0.97.

#### 2.4.4. Parental Child-Containing Function Scale (PCCFS)

The scale consists of a list of 36 items based on parental–child clinical observations and Bion’s [13] concept of inclusive function. It was developed by Zabçı, Erol, and Şimşek [17], and its Cronbach’s alpha value was found to be 0.81. In this study, the Cronbach’s alpha value for the scale was calculated as 0.90.

#### 2.4.5. Dissociative Experiences Scale (DES)

The Dissociative Experiences Scale is a 28-item self-report scale used to screen and rate alienation from oneself and the environment [26]. The Turkish validity and reliability study of the scale was carried out by Yargıç, Tutkun, and Şar [27], and the Cronbach alpha value was calculated as 0.91. In this study, the Cronbach’s alpha value for the scale was calculated as 0.95.

## 3. Results

The results of the regression analysis regarding the mediating role of dissociative experiences in the effect of physical abuse and neglect on emotion regulation difficulties are presented in Table 2. When Table 2 is examined, it was found that physical abuse significantly predicted dissociative experiences with a mediating variable (β = 0.324; 95% CI [2.897; 5.236]; *p* < 0.01), while dissociative experiences with a mediating variable predicted difficulty in emotion regulation in a positive way (β = 0.608; 95% CI [0.202; 0.263]; *p* < 0.01). In addition, the overall effect of physical abuse on the dependent variable, difficulty in emotion regulation, was found to be significant (β = 0.300; 95% CI [0.990; 1.892]; *p* < 0.01). In addition, when physical abuse and dissociative experiences are included in the regression analysis together, it is seen that the effect of physical abuse on difficulty in emotion regulation decreases (β = 0.103; 95% CI [0.114; 0.875]; *p* < 0.01). It was observed that the indirect effect of physical abuse on emotion regulation difficulties through dissociative experiences was significant (β = 0.197; 95% CI [0.124; 0.268]). As with physical abuse, physical neglect significantly predicted dissociative experiences with the mediating variable (β = 0.428; 95% CI [1.384; 2.028]; *p* < 0.01), while dissociative experiences with the mediating variable significantly predicted difficulty in emotion regulation (β = 0.543; 95% CI [0.177; 0.238]; *p* < 0.01). In addition, the overall effect of physical neglect on the dependent variable, difficulty in emotion regulation, was found to be significant (β = 0.462; 95% CI [1.384; 1.028]; *p* < 0.01). In addition, when physical neglect and dissociative experiences were included in the regression analysis together, the effect of physical neglect on emotional dysregulation decreased (β = 0.230; 95% CI [0.551; 1.145]; *p* < 0.01), physical neglect had a greater effect on difficulty in emotion regulation, and the indirect effect through self-dissociative experiences was significant (β = 0.232; 95% CI [0.161; 0.306]). When these findings were evaluated, it was seen that dissociative experiences had an indirect effect on the effect of physical abuse and neglect on emotional dysregulation.

An analysis of the mediating role of dissociative experiences in the effects of emotional abuse and emotional neglect on emotional dysregulation and parental child-containing function is presented in Table 3. When Table 3 is examined, emotional abuse significantly predicted dissociative experiences with the mediator variable (β = 0.499; 95% CI [3.304; 4.667]; *p* < 0.01), while dissociative experiences with the mediating variable significantly predicted difficulty in emotion regulation (β = 0.476; 95% CI [0.151; 0.213]; *p* < 0.01). In addition, the overall effect of emotional abuse on the dependent variable, difficulty in emotion regulation, was found to be significant (β = 0.568; 95% CI [1.489; 1.984]; *p* < 0.01). However, when emotional abuse and dissociative experiences were included together in the regression analysis, it was observed that the effect of emotional abuse on emotional dysregulation decreased (β = 0.238; 95% CI [0.762; 1.257]; *p* < 0.01). It was observed that the indirect effect of emotional abuse on emotion regulation difficulties through dissociative experiences was significant (β = 0.238; 95% CI [0.169; 0.309]). In addition, it was observed that emotional neglect significantly predicted dissociative experiences with the mediating variable (β = 0.435; 95% CI [2.573; 3.834]; *p* < 0.01), while dissociative experiences significantly predicted difficulty in emotion regulation (β = 0.483; 95% CI [0.155; 0.213]; *p* < 0.01). In addition, the overall effect of emotional neglect on the dependent variable, difficulty in emotion regulation, was significant (β = 0.575; 95% CI [1.383; 1.834]; *p* < 0.01), when emotional neglect and dissociative experiences were included in the regression analysis together, the effect of emotional neglect on emotion regulation was found to be significant. It seems that the effect on disability decreased (β = 0.364; 95% CI [0.807; 1.232]; *p* < 0.01). The indirect effect of emotional neglect through dissociative experiences on difficulty in emotion regulation was found to be significant (β = 0.210; 95% CI [0.150; 0.275). When these findings were evaluated together, it was seen that dissociative experiences had an indirect effect on the effect of emotional abuse and emotional neglect on emotion regulation difficulties.

The results of the regression analysis regarding the mediating role of dissociative experiences in the effect of sexual abuse on emotional dysregulation are presented in Table 4. When the results of the analysis are evaluated, it was seen that dissociative experiences, which were the mediator variable of sexual abuse, were positively significant (β = 0.222; 95% CI [1.655; 4.164]; *p* < 0.01), while dissociative experiences significantly predict difficulty in emotion regulation (β = 0.626; 95% CI [0.210; 0.269]; *p* < 0.01). In addition, the overall effect of sexual abuse on dysregulation was significant (β = 0.209; 95% CI [0.564; 1.527]; *p* < 0.01). However, when sexual abuse and dissociative experiences were included together in the regression analysis, the effect of sexual abuse on dysregulation seems to have disappeared (β = 0.069; 95% CI [−0.037; 0.735]; *p* > 0.05). It was observed that the indirect effect of sexual abuse through dissociative experiences on emotional dysregulation was significant (β = 0.139; 95% CI [0.058; 0.220]). The findings show that dissociative experiences had an indirect effect on the effect of sexual abuse on emotional dysregulation.

The results of the regression analysis regarding the mediating role of dissociative experiences in the effect of physical abuse and physical neglect on parental child-containing function are presented in Table 5. When the analysis results were examined, it was found that physical abuse significantly predicted dissociative experiences with mediator variable (β = 0.185; 95% CI [0.456; 1.455]; *p* < 0.01), while dissociative experiences predicted parental child-containing function positively. (β = 0.379; 95% CI [0.116; 0.195]; *p* < 0.01). In addition, the overall effect of physical abuse on the dependent variable, parental child-containing function, was found to be significant (β = 0.185; 95% CI [0.456; 1.455]; *p* < 0.01). However, when physical abuse and dissociative experiences were included in the regression analysis together, the effect of physical abuse on parental child-containing function disappeared (β = 0.062; 95% CI [−0.170; 0.814]; *p* > 0.05), and physical abuse was found to be related to parental child-containing function. The indirect effect of dissociative experiences on inclusive function was found to be significant (β = 0.122; 95% CI [0.071; 0.181]). Physical neglect also significantly predicted dissociative experiences (β = 0.428; 95% CI [3.268; 4.985]; *p* < 0.01), while dissociative experiences significantly predicted parental child-containing function (β = 0.352; %95 CI [0.104; 0.186]; *p* < 0.01). In addition, the overall effect of physical neglect on the dependent variable, parental child-containing function was found to be significant (β = 0.259; 95% CI [0.652; 1.406]; *p* < 0.01). When physical neglect and dissociative experiences were included in the regression analysis together, the effect of physical neglect on parental child-containing function decreased (β = 0.108; 95% CI [0.104; 0.186]; *p* < 0.01), and physical neglect had a negative impact on parental child-containing function. The indirect effect through experiences was found to be significant (β = 0.151; 95% CI [0.084; 0.228]). When these findings were evaluated together, it was seen that dissociative experiences had an indirect effect on the effect of physical abuse and physical neglect on parental child-containing function.

Table 6 shows the results of the regression analysis regarding the mediating role of dissociative experiences in the effects of emotional abuse and emotional neglect on parental child-containing function. When the results of the analysis are examined, it was seen that emotional abuse significantly predicted dissociative experiences with the mediator variable (β = 0.499; 95% CI [3.304; 4.667]; *p* < 0.01), while dissociative experiences significantly predicted parental child-containing function (β = 0.316; 95% CI [0.087; 0.172]; *p* < 0.01). In addition, the overall effect of emotional abuse on the parental child-containing function, which was the dependent variable, was found to be significant (β = 0.324; 95% CI [0.758; 1.370]; *p* < 0.01). However, when emotional abuse and dissociative experiences were included in the regression analysis together, the effect of emotional abuse on parental child-containing function decreased (β = 0.166; 95% CI [0.207; 0.884]; *p* < 0.01). The indirect effect of dissociative experiences on function was found to be significant (β = 0.158; 95% CI [0.086; 0.238]). Considering the results of the data on emotional neglect, it is seen that emotional neglect significantly predicts dissociative experiences (β = 0.435; 95% CI [2.573; 3.834]; *p* < 0.01), while dissociative experiences significantly predict parental child-containing function (β = 3.834). 0.366; 95% CI [0.109; 0.192]; *p* < 0.01). The total effect of emotional neglect on parental child-containing function, which was the dependent variable, was significant (β = 0.234; 95% CI [0.418; 0.994]; *p*< 0.01), when emotional neglect and dissociative experiences were included together in the regression analysis, the effect of emotional neglect on parental child-containing function. It was observed that the effect of emotional neglect on parental child-containing function disappeared (β = 0.075; 95% CI [−0.075; 0.527]; *p* > 0.05) and the indirect effect of emotional neglect on parental child-containing function through dissociative experiences was significant (β = 0.159; 95% CI [0.093; 0.235]). When the findings were evaluated together, it was seen that dissociative experiences had an indirect effect on the effect of emotional abuse and emotional neglect on parental child-containing function.

The results of the regression analysis regarding the mediating role of dissociative experiences in the effect of sexual abuse on parental child-containing function are presented in Table 7. It was observed that sexual abuse significantly predicted dissociative experiences with mediator variable (β = 0.222; 95% CI [1.655; 4.164]; *p* < 0.01), while dissociative experiences significantly predicted parental child-containing function (β = 4.164). 0.387; 95% CI [0.121; 0.197]; *p* < 0.01). In addition, the overall effect of sexual abuse on parental child-containing function, which is the dependent variable, was found to be significant (β = 0.139; 95% CI [0.225; 1.273]; *p* < 0.01). When sexual abuse and dissociative experiences were included in the regression analysis together, the effect of sexual abuse on parental child-containing function disappeared (β = 0.053; 95% CI [−0.212; 0.783]; *p* > 0.05), and that sexual abuse had a greater impact on parental child-containing function. The indirect effect through dissociative experiences was found to be significant (β = 0.086; 95% CI [0.039; 0.150]). These findings show that dissociative experiences indirect effects of sexual abuse on parental child-containing function.

## 4. Discussion

The results of the research can be grouped under two headings. The first title shows that dissociative experiences have an indirect effect on the effect of childhood traumas (physical abuse and physical neglect, emotional abuse and emotional neglect and sexual abuse) on emotion regulation difficulties. The second title of the research results shows that dissociative experiences have an indirect effect of childhood traumas (physical abuse and physical neglect, emotional abuse and emotional neglect and sexual abuse) on parental child-containing function. When the results of the study are evaluated together, it can be said that dissociation has an indirect effect on both mood regulation skills and parental child-containing function skills in individuals with childhood traumatic experiences.

The research findings are supported by the literature. When the literature is examined, dissociation has a protective function against intense stress resulting from trauma [28] and it is mediated by dissociation in trauma and emotion regulation [29], it can be seen that the integrity of the components of emotions in individuals who have been exposed to a traumatic life event was broken and it has been reported that individuals exposed to trauma during childhood have difficulty in regulating their emotions harmoniously and perceive their emotion regulation less successfully than controls who have never been exposed to trauma [30,31,32,33]. In addition, abused children exhibit less appropriate emotions, decreased empathy and emotional self-awareness, and show more emotional instability or negativity [34]. A history of childhood trauma history and a diagnosis of difficulty regulating emotion are associated with each other [35], and the relationship between childhood trauma and psychopathology is mediated by emotion regulation abilities; the inability to regulate emotions harmoniously is one of the most distinctive features of being exposed to trauma [36]. It has been reported that emotional abuse and neglect may be as important as physical and sexual abuse in the development of dissociative symptoms [37]. In a study conducted by Chu [38], with 90 individuals treated in the department of trauma and dissociative disorders, it was found that the majority of these patients were abused in the pre-adolescent period; 83% of the patients were physically abused, 82% were sexually abused, and 64% reported violence.

Exposure to childhood abuse and neglect experiences increases the risk of experiencing dissociative symptoms in adulthood and that these individuals may display shy, cowardly, and antisocial behaviors in their relationships with others, and that children develop trauma-specific behavior patterns (quick reaction, avoidance, helplessness, destructive behaviors) in abusive experiences and emerge. The information that they can carry, i.e., their emerging schemas, to adult life [39,40] is available in the literature. While Freud’s early articles focused on high-impact physical or sexual childhood trauma, the recent psychoanalytic literature has highlighted the impact of perceived or actual chronic emotional abuse and neglect on the developing personality, leading to failures in the mother’s capacity to “mentalize” her child in her own mind [41]. A child may exhibit dissociative responses as a developmental necessity to keep the self free from potential collapse and fragmentation in response to high anxiety or overstimulation not only caused by intrusive trauma, but also by the failure of parents to provide the child with an emotionally inclusive environment. Repeated emotional trauma can then have a cumulative effect, facilitating the tendency to dissociate under stress and become a habit [42]. Moreover, it is stated that dissociative parental behaviors are more common in individuals with a traumatic past [43]. Exposure to continuous, repetitive or multiple traumas in childhood not only causes post-traumatic stress symptoms, but also predominantly impacts emotional and interpersonal self-esteem. It has been suggested that this results in a complex symptom presentation, including other symptoms reflecting impairments in regulatory capacities. Anxious arousal, anger management, dissociation symptoms, and difficulties with aggressive, or social avoidance behaviors are just some of them [44]. This can play a disruptive role in parental child-containing function skills. When a parent with dissociative symptoms with a traumatic experience in childhood cannot adequately respond to an emotional response from the child during the child’s development, the child may not learn self-control (self-regulation) or the ability to inhibit inappropriate emotional responses (self-control).

This research has some limitations. Data were collected through online applications. Accordingly, participants may have been affected by personal and environmental conditions while filling out the scales. It is recommended to collect data face-to-face in future studies. Another limitation is that filling out the online scales on the internet has brought about the limitation of reaching the mothers who do not have access to the Internet. Giving sampling scales through face-to-face interviews can remove this limitation. Another limitation is that the data are based on the answers of the participants. Because data-collection tools are self-report scales. In future research, the use of clinical evaluation principles (clinical interview, psychological tests, case studies, etc.) in addition to self-report scales may play a role in increasing the validity of the data to be obtained. As another limitation is that the lack of longitudinal temporal sequence to the outcome, the mediators and the predictors.

## 5. Conclusions

In mothers exposed to childhood trauma, dissociative experiences play a mediating role in emotional regulation skills and parental child-containing function of mothers and dissociative experiences having an indirect effect on these skills.

## Figures and Tables

**Table 1:** Demographic Characteristics of Participants.

Variable	Groups	n	%
Age	20–30 Years	33	8.3
	31–40 Years	200	50.0
	41–50 Years	136	34.0
	51–60 Years	31	7.8
Educational Status	Primary school	21	5.3
	Middle school	16	4.0
	High school	78	19.5
	University	207	51.7
	Postgraduate/PhD	78	19.5
Marital status	Single	4	1.0
	Married	355	88.8
	Divorced	36	9.0
	Spouse Deceased/Living Alone	5	1.3
	Living Together	4	1.0
Working Status	Housewife	145	36.3
	Part time	34	8.5
	There is order work	221	55.3
Number of children she has	One Child	122	30.5
	Two kids	193	48.3
	Three Children	61	15.3
	Four and More	24	5.9
Relationship Status with Her Child	Very good	180	45.0
	Good	188	47.0
	Middle	30	7.5
	Bad	2	0.5
	Too bad	0	0.0
Perceived Income Level	Bad	199	49.7
	Middle	77	19.3
	Good	124	31.0
History of receiving psychological support	Yes	172	43.0
	No	228	57.0
Having a Migration History	Yes	213	53.3
	No	187	46.8

**Table 2:** Results of Regression Analysis Regarding the Mediator Role of Dissociative Experiences in the Effect of Physical Abuse and Neglect on Emotion Regulation Difficulty.

Dependent Variable	Independent Variable	B	Standard Error	Beta	t	*p*	Tolerance	VIF	Residual Statistic
Dissociative Experiences	Physical Abuse	4.066	0.594	0.324	6.836	0.000 **	0.730	1.88	16.33
	R = 0.234	R^2^ = 0.054		Sd:1/398	F: 14.218		*p* = 0.000 **		
Difficulty Regulating Emotion	Physical Abuse	0.495	0.193	0.103	2.557	0.010 **	8.10	1.23	16.12
	Dissociative Experiences	0.232	0.015	0.608	15.076	0.000 **	8.33	1.33	
	R = 0.649	R^2^ = 0.421		Sd:2/397	F: 9.311		*p* = 0.000 **		
Difficulty Regulating Emotion	Physical Abuse	1.114	0.229	0.300	1.441	0.000 **	9.22	1.44	16.10
	R = 0.300	R^2^ = 0.090		Sd:1/398	F: 39.485		*p* = 0.000 **		
Dissociative Experiences	Physical Neglect	4.127	0.436	0.428	9.450	0.000 **	.7.44	1.77	16.02
	R = 0.428	R^2^ = 0.183		Sd:1/398	F: 89.318		*p* = 0.000 **		
Difficulty Regulating Emotion	Physical Neglect	0.848	0.151	0.230	10.023	0.010 **	7.33	1.23	16.08
	Dissociative Experiences	0.207	0.015	0.543	13.255	0.000 **			
	R = 0.674	R^2^ = 0.455		Sd:2/397	F: 165.831		*p* = 0.000 **		
Difficulty Regulating Emotion	Physical Neglect	1.706	0.163	0.462	10.411	0.000 **	7.99	1.22	16.09
	R = 0.462	R^2^ = 0.323		Sd:1/398	F: 108.389		*p* = 0.000 **		

** *p* < 0.01.

**Table 3:** Results of Regression Analysis Regarding the Mediator Role of Dissociative Experiences in the Effect of Emotional Abuse and Neglect on Emotion Regulation Difficulty.

Dependent Variable	Independent Variable	B	Standard Error	Beta	t	*p*
Dissociative Experiences	Emotional Abuse	3.986	0.346	0.499	11.500	0.000 **
R = 0.499	R^2^ = 0.249	Sd:1/398	F: 132.252		*p* = 0.000 **	
Difficulty Regulating Emotion	Emotional Abuse	1.010	0.125	0.330	8.027	0.010 **
	Dissociative Experiences	0.182	0.015	0.476	11.567	0.000 **
R = 0.702	R^2^ = 0.494	Sd:2/397	F: 193.843		*p* = 0.000 **	
Difficulty Regulating Emotion	Emotional Abuse	1.736	0.125	0.568	13.796	0.000 **
R = 0.568	R^2^ = 0.323	Sd:1/398	F: 190.353		*p* = 0.000 **	
Dissociative Experiences	Emotional Neglect	3.185	0.330	0.435	9.653	0.000 **
R = 0.435	R^2^ = 0.189	Sd:1/398	F: 93.193		*p* = 0.000 **	
Difficulty Regulating Emotion	Emotional Neglect	1.020	0.108	0.364	9.435	0.010 **
	Dissociative Experiences	0.184	0.014	0.483	12.499	0.000 **
R = 0.720	R^2^ = 0.519	Sd:2/397	F: 214.755		*p* = 0.000 **	
Difficulty Regulating Emotion	Emotional Neglect	1.608	0.114	0.575	14.020	0.000 **
R = 0.575	R^2^ = 0.330	Sd:1/398	F: 175.388		*p* = 0.000 **	

** *p* < 0.01.

**Table 4:** Results of Regression Analysis Regarding the Mediator Role of Dissociative Experiences in the Effect of Sexual Abuse on Emotion Regulation Difficulty.

Dependent Variable	Independent Variable	B	Standard Error	Beta	t	*p*
Dissociative Experiences	Sexual Abuse	2.910	0.638	0.222	4.560	0.000 **
Difficulty Regulating Emotion	Sexual Abuse	0.349	0.196	0.069	1.776	0.010 **
	Dissociative Experiences	0.239	0.150	0.623	15.925	0.000 **
Difficulty Regulating Emotion	Sexual Abuse	1.046	0.244	0.209	4.272	0.000 **

** *p* < 0.01.

**Table 5:** Regression Analysis Results on the Mediator Role of Dissociative Experiences in the Effect of Physical Abuse and Neglect on Parental Child-Containing Function.

Dependent Variable	Independent Variable	B	Standard Error	Beta	t	*p*
Dissociative Experiences	Physical Abuse	4.066	0.594	0.324	6.836	0.000 **
R = 0.324	R^2^ = 0.105	Sd:1/398	F: 46.743		*p* = 0.000 **	
Parental Child-Containing Function	Physical Abuse	0.322	0.250	0.062	1.285	0.199
	Dissociative Experiences	0.155	0.0200	0.397	7.810	0.000 **
R = 0.403	R^2^ = 0.163	Sd:2/397	F: 38.645		*p* = 0.000 **	
Parental Child-Containing Function	Physical Abuse	0.956	0.254	0.185	3.762	0.000 **
R = 0.185	R^2^ = 0.034	Sd:1/398	F: 14.153		*p* = 0.000 **	
Dissociative Experiences	Physical Neglect	4.127	0.436	0.428	9.450	0.000 **
R = 0.428	R^2^ = 0.183	Sd:1/398	F: 89.318		*p* = 0.000 **	
Parental Child-Containing Function	Physical Neglect	0.430	0.200	0.180	2.145	0.032 *
	Dissociative Experiences	0.145	0.028	0.352	6.970	0.000 **
R = 0.411	R^2^ = 0.169	Sd:2/397	F: 40.401		*p* = 0.000 **	
Parental Child-Containing Function	Physical Neglect	1.029	0.191	0.259	5.364	0.000 **
R = 0.259	R^2^ = 0.067	Sd:1/398	F: 28.778		*p* =0.000 **	

** *p* < 0.01, * *p* < 0.05.

**Table 6:** Regression Analysis Results on the Mediator Role of Dissociative Experiences in the Effect of Emotional Abuse and Neglect on Parental Child-Containing Function.

Dependent Variable	Independent Variable	B	Standard Error	Beta	t	*p*
Dissociative Experiences	Emotional Abuse	3.986	0.346	0.499	11.500	0.000 **
R = 0.499	R^2^ = 0.249	Sd:1/398	F: 132.252		*p* = 0.000 **	
Parental Child-Containing Function	Emotional Abuse	0.545	0.172	0.166	3.168	0.199
	Dissociative Experiences	0.130	0.021	0.316	6.031	0.000 **
R = 0.424	R^2^ = 0.480	Sd:2/397	F: 43.634		*p* = 0.000 **	
Parental Child-Containing Function	Emotional Abuse	1.064	0.155	0.324	6.836	0.000 **
R = 0.324	R^2^ = 0.105	Sd:1/398	F: 46.736		*p* = 0.000 **	
Dissociative Experiences	Emotional Neglect	3.185	0.330	0.435	9.653	0.000 **
R = 0.435	R^2^ = 0.189	Sd:1/398	F: 93.193		*p* = 0.000 **	
Parental Child-Containing Function	Emotional Neglect	0.226	0.153	0.075	1.470	0.140
	Dissociative Experiences	0.150	0.021	0.366	7.190	0.000 **
R = 0.405	R^2^ = 0.164	Sd:2/397	F: 38.958		*p* = 0.000 **	
Parental Child-Containing Function	Emotional Neglect	0.706	0.146	0.234	4.821	0.000 **
R = 0.234	R^2^ = 0.055	Sd:1/398	F: 23.248		*p* = 0.000 **	

** *p* < 0.01.

**Table 7:** Regression Analysis Results Regarding the Mediator Role of Dissociative Experiences in the Effect of Sexual Abuse on Parental Child-Containing Function.

Dependent Variable	Independent Variable	B	Standard Error	Beta	t	*p*
Dissociative Experiences	Sexual Abuse	2.910	0.638	0.222	4.560	0.000 **
R = 0.222	R^2^ = 0.049	Sd:1/398	F: 20.799		*p* = 0.000 **	
Parental Child-Containing Function	Sexual Abuse	0.285	0.253	0.053	1.128	0.259
	Dissociative Experiences	0.159	0.019	0.387	8.222	0.000 **
R = 0.402	R^2^ = 0.136	Sd:2/397	F: 38.420		*p* = 0.000 **	
Parental Child-Containing Function	Sexual Abuse	0.749	0.266	0.139	2.811	0.000 **
R = 0.139	R^2^ = 0.019	Sd:1/398	F: 7.902		*p* = 0.000 **	

** *p* < 0.01.

## Data Availability

The data presented in this study are available on request from the corresponding author. The data are not publicly available due to privacy.
